# Temperament Traits and Psychopathology in Young Clinically Referred Children Compared to a General Population Sample

**DOI:** 10.1007/s10578-016-0708-6

**Published:** 2017-01-18

**Authors:** Frederike Y. Scheper, Mirjana Majdandžić, Peter M. van de Ven, Lucres M. C. Jansen, Theo A. H. Doreleijers, Carlo Schuengel, Annelou L. C. de Vries

**Affiliations:** 1 Department of Child and Adolescent Psychiatry, VU University medical centre, Amsterdam, The Netherlands; 2 Research Institute of Child Development and Education, University of Amsterdam, Amsterdam, The Netherlands; 3 Department of Epidemiology and Biostatistics, VU University medical centre, Amsterdam, The Netherlands; 4 Section of Clinical Child and Family Studies, Vrije Universiteit Amsterdam, Amsterdam, The Netherlands

**Keywords:** Temperament, Internalizing and externalizing behavior problems, Behavior disorders, Early childhood, Clinically referred

## Abstract

Evidence from general population studies shows the contribution of various temperament traits to the development of child psychopathology. Little is known about which traits are associated with internalizing and externalizing problems in young clinically referred children. The current study assessed temperament and internalizing and externalizing problems in 216 referred children (*M* = 4.35 years, *SD* 0.89, 81% boys). A comparison was made with an age and gender matched general population sample. Referred children showed less effortful control than general population children. Less effortful control and more negative affectivity were associated with more internalizing and externalizing problems across groups. Surgency, and specifically temperamental impulsivity, was more strongly associated with externalizing problems in referred children compared to general population. Less soothability, less inhibitory control and more frustration predicted (sub)clinical levels of comborbid internalizing and externalizing problems in referred children. The results can be used in diagnostic and treatment procedures in early childhood.

## Introduction

Prevalence rates of behavioral and emotional problems and disorders in early childhood are similar to prevalence rates in older children [1–4]. Based on the recognition of the mental health needs of young children [5], clinical referral options are now available in many countries. Specific temperament traits, defined as constitutionally based differences in emotional reactivity and self-regulation [6], have been found to predict behavioral (externalizing) problems and emotional (internalizing) problems in early childhood in several general population studies [7–9]. Studies on links between temperament traits and internalizing and externalizing problem behavior in young clinically referred children are scarce, however. We therefore do not know to what extent these associations are similar across general populations and clinically referred populations. Therefore, this study investigated temperament traits and their associations with internalizing and externalizing problem behavior in young clinically referred children and compared the strenghts of these associations to an age and gender matched general population sample.

Temperament is largely seen as the expression of heritable characteristics as these unfold through maturation and experience [10]. Neural networks including the prefrontal and anterior cingulate cortices develop under influence of genetic and environmental factors and play an important role in emotional reactivity but also in emotion regulation, attention, and cognitive control [11, 12]. In young children, age three through seven, three broad temperament dimensions have been identified by Rothbart and colleagues: negative affectivity, extraversion/surgency and effortful control [13, 14]. The first two dimensions, negative affectivity and surgency, represent the tendency of children to react with either negative or positive emotions to daily situations. Children high in negative affectivity respond more readily with fear, sadness and/or anger and frustration in situations, while children with high surgency are inclined to express laughter, impulsivity, activity and approach. The third dimension, effortful control (self-regulation), represents the ability to voluntarily regulate behavioral reactivity and attention, expressed by the inhibition of a dominant response and activation of a subdominant response [15].

Several models have been proposed to explain the association between temperament and psychopathology [16]. There is evidence for a spectrum model, which proposes that temperament traits and psychiatric disorders share etiological factors and vary along the same continuum with extreme levels of temperament traits considered psychopathology [17–19]. There is also evidence for a vulnerability or resilience model, in which specific temperament traits predispose towards or protect against the onset of psychopathology in specific contexts [20]. Furthermore, there are models in which temperament influences the expression of psychiatric symptoms (pathoplasty model), and vice versa, that psychopathology influences the expression of temperament (scar model) [16, 17, 21]. These last two theoretical models imply that associations between temperament traits and psychopathology could be different in clinically referred children with emotional and behavioral problems compared to non-referred children in the general population.

In population studies, there is consistent evidence that high levels of negative affectivity and more fine-grained traits within this dimension (such as frustration, sadness, fear and low levels of soothability) predict both externalizing and internalizing problems in infancy, preschool age, and school age [7, 22–25]. Low levels of effortful control and more fine-grained traits within this dimension (such as attention focusing, inhibitory control and low-intensity pleasure) were found to predict externalizing problem behavior, also when internalizing problems co-occurred [22, 24]. Low levels as well as high levels of effortful control have been found associated with internalizing problems in general population children [26]. High levels of impulsivity and activity level (subscales of surgency) were found to predict externalizing problems at preschool and school age, whereas shyness was associated with internalizing problems [22, 24]. In sum, there is evidence from several general population studies that different temperament traits are associated to both internalizing and externalizing problems and there is also evidence for specific temperament traits predicting specific problem behavior. In addition, there is evidence from population studies for trait-by-trait moderation, such that negative affectivity is most strongly predicting externalizing problem behavior when effortful control is low [24, 26].

Results from studies linking temperament to child problem behavior have given rise to preventive interventions in the general population. These interventions focus on increasing parents’ and teachers’ understanding of their child’s temperament, providing tools to deal with the child’s temperament traits and modifying children’s patterns of behavior such as increasing children’s self-regulation [27, 28]. Clinically referred children already display problematic behavior which could, directly or indirectly, influence their temperament traits as perceived by their parents. However, in contrast to studies in general population children, there are few studies on the relation between temperament traits and internalizing and externalizing problem behavior in young clinically referred children. Also, comparison of studies and interpreting results is difficult due to differences on the definitions of temperament. In a small clinically referred sample of preschool children, high levels of negative affectivity were found to be associated with symptoms of anxiety, reflecting internalizing problems [29], but no comparison was made with general population children.

In this study, we therefore investigated the levels of temperament traits (as defined by Rothbart et al. [14]) and the associations between temperament and problem behavior in young children referred for mental health care. We compared this to an age and gender matched general population sample. First, we compared referred children with general population children on levels of broad temperament dimensions and fine-grained temperament traits. We expected that referred children display higher levels of negative affectivity and lower levels of effortful control (and related fine-grained traits) than general population children. Second, we examined associations between temperament traits and problem behavior. The associations between the three broad temperament dimensions, alone and in interaction, and internalizing and externalizing problems were examined in a path model in which all relations were estimated simultaneously. Possible differences in associations between clinically referred children and general population children were determined. Furthermore, we examined which fine-grained temperament traits were associated with internalizing problems and with externalizing problems in clinically referred children and whether these associations differed from those in general population children.

## Methods

### Participants

The clinically referred sample consisted of 216 children, age 3.00–7.33 (*M* 4.35, *SD* 0.89), 81% boys. For comparison with general population children, a subsample of 115 referred children was matched on age and gender, and as much as possible on ethnicity and IQ, with a sample of 115 non-referred children, aged 3.00–7.42 years (*M* = 4.26 years, *SD* 0.99), 65% boys. Sample characteristics are displayed in Table 1. In the clinically referred group there were significant more children with non-western ethnicity than in the non-referred general population group, although birth countries were missing especially in the population sample [24 (21%) missings compared to 2 (2%) in the referred sample].

**Table 1:** Descriptives and comparison of clinically referred (N = 115) and general population children (N = 115)

	Referred *N* = 115	Population *N* = 115	*χ* ^*2*^/*t*	*p*
Gender, *N* (%)				
Male	75 (65)	75 (65)	0.00	1.000
Age, *M* (*SD*)				
Age in years	4.26 (0.99)	4.26 (0.99)	0.03	.979
Ethnicity, *N* (%)				
Non-western origin^a^	27 (24%)	9 (10%)	6.80	.009
Child problem behavior, *M* (*SD*)				
Internalizing problem behavior	61.4 (8.64)	46.2 (10.25)	−11.95	<.001
Externalizing problem behavior	62.0 (11.02)	48.8 (10.37)	−9.18	<.001

^a^Non-Western origin was defined as: one or more biological parents born in Africa, Turkey, Latin America or Asia, excluding Indonesia and Japan, conform the criteria of Statistics Netherlands

The clinically referred children were recruited when they were assessed for outpatient treatment of emotional and/or behavioral problems to MOC ‘t Kabouterhuis, a health care institution for early childhood in Amsterdam, the Netherlands. The non-referred general population sample was derived from a large longitudinal study on temperament in early childhood in Amsterdam, the Netherlands [30]. Families were recruited through birth records of the Municipal Health Service of Amsterdam; families received tickets for the local zoo after completing the first measurement occasion.

### Procedure

Parents completed questionnaires on child temperament and internalizing and externalizing problem behavior. The questionnaires in the referred group were completed by the mothers, who were the primary caregivers. In the population study, questionnaires were available of fathers and mothers, but only mothers’ reports were used in the current study for comparison with the referred group.

Caregivers of the clinically referred children signed informed consent for using data for scientific research and knew that participation in the study would not influence treatment. The study on the clinically referred group was approved by the medical ethical committee of the VU University Medical Center in Amsterdam. The population study was approved by the ethical committee of the Municipal Health Service of Amsterdam.

### Measures

#### Child Temperament

The Children’s Behavior Questionnaire (CBQ; [13, 14]) was used to assess temperament dimensions and fine-grained temperament traits of the children. In the general population group, the original long 195-item version of the CBQ was used, and in the clinically referred group, the 94-item short version. Therefore, only the items of the short version were used from the data of the population study. The CBQ short-form is a parent-report instrument containing 94 items referring to concrete occurrences of child reactivity and regulation in commonly occurring situations (e.g., frustration: “my child gets angry when told he or she needs to go to bed”, inhibitory control: “my child can wait until entering into new activities if (s)he is asked to”). Ratings, on a 7-point Likert scale, refer to the degree to which the statement applies to the child (from 1 = extremely untrue to 7 = extremely true). Earlier research using the CBQ short-form demonstrates satisfactory internal consistency and criterion validity and longitudinal stability [13]. In previous research using the Dutch translation of the CBQ, a principal axis factor analysis provided evidence for the three temperament dimensions negative affectivity, surgency and effortful control [30]. Negative affectivity included the fine-grained traits: anger/frustration, discomfort, sadness and (reversed) soothability. Surgency included the fine-grained traits: activity level, approach, high-intensity pleaure, impulsivity, shyness (reversed) and smiling/laughter. Effortful control included: attention focusing, inhibitory control, low-intensity pleasure and perceptual sensitivity. In the current study, internal consistency at both levels of temperament was satisfactory. Internal consistency (Cronbach’s alphas across items for the total group) of the dimensions was 0.69 for negative affectivity, 0.63 for surgency and 0.71 for effortful control. The internal consistency (Cronbach’s alphas) for the fine-grained traits was 0.72 for anger/frustration, 0.71 for discomfort, 0.60 for fear, 0.63 for sadness, 0.85 for soothability, 0.70 for activity level, 0.72 for approach, 0.74 for high intensity pleaure, 0.70 for impulsivity, 0.84 for shyness (reversed), 0.67 for smiling/laughter, 0.81 for attention focusing, 0.68 for inhibitory control, 0.69 for low intensity pleasure and 0.75 for perceptual sensitivity.

#### Child Problem Behavior

To assess internalizing and externalizing problem behavior, caregivers of the clinically referred children completed the Child Behavior Checklist (CBCL), for ages 1.5–5 [31] or ages 6–18 [32]. Caregivers of the children recruited in the population study completed the CBCL for ages 2–3 or ages 4–18 [33], as this study was conducted before the new CBCL versions were available. Caregivers were asked to rate items on a three point scale (0 = not at all true, 1 = somewhat true, 2 = very true). In this study we used the T-scores of the broadband syndrome scales internalizing and externalizing problem behavior. T-scores were computed to correct for age and gender and to make the scores between the two study groups comparable. The T-scores of the broadband syndrome scales internalizing and externalizing problem behavior were computed when no more than four items were missing on internalizing behavior and no more than three items were missing on externalizing behavior. This led to exclusion of eight cases; four in the referred group and four in the population group. In the referred group, the internal consistency (Cronbach’s alphas) for the CBCL 1.5–5 (*N* = 208) was 0.72 for internalizing problems (across four small band scales) and 0.72 for externalizing problems (across two small band scales). The internal consistency of the CBCL 6–18 could not be determined because of small number of participants (*N* = 9). In the general population group, the internal consistency (Cronbach’s alphas) for the CBCL 2–3 (*N* = 37) was 0.88 for internalizing problems (25 items) and 0.90 for externalizing problems (26 items) and for the CBCL 4–18 (*N* = 73) 0.80 for internalizing problems (31 items) and 0.87 for externalizing problems (33 items).

### Data Analysis Plan

First, mean scores on temperament dimensions and scales were compared between the referred and population group using independent samples *t* tests. To account for multiple testing (18 tests), we used the Bonferroni correction and considered significant only those temperament traits for which the *p* < .05/18 = 0.0028.

**Fig. 1:**
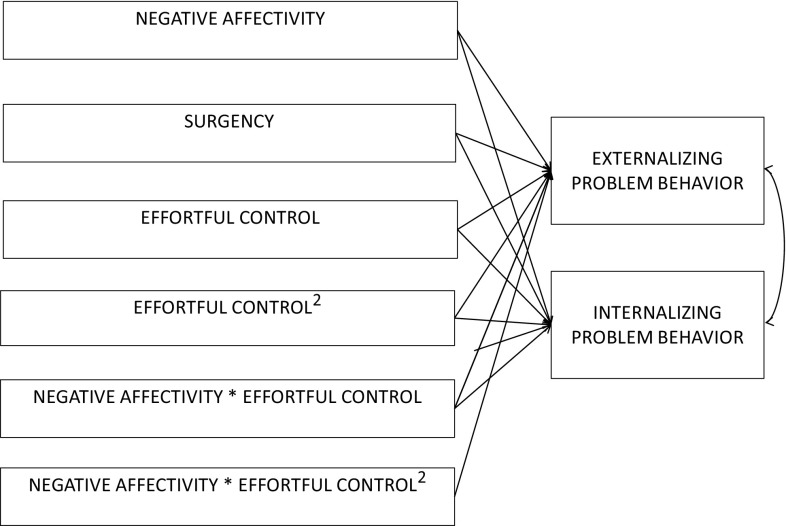
The path model of temperament dimensions in relation to child problem behavior in referred children (N = 115) and general population children (N = 115)

Second, relations between the broad temperament dimensions (negative affectivity, surgency, and effortful control) and externalizing and internalizing problem behaviors were examined and compared between clinically referred and general population children by using multigroup path analyses in M-plus 6.11 [34]. The path model, in which all relations are estimated simultaneously, included internalizing and externalizing problems as dependent variables. The independent variables were the three broad temperament dimensions plus a quadratic term for effortful control, and interactions between negative affectivity and the linear and quadratic term for effortful control. The quadratic term for effortful control was included because both low as well as high levels of effortful control have been found to be associated with child internalizing problem behavior in population studies. The interactions between effortful control and negative affectivity were included in order to examine the role of effortful control as a possible moderator of the relation between negative affectivity and child problem behavior. All temperament variables were mean centered to facilitate interpretation of the interaction terms. A path model was fitted allowing coefficients for paths between independent and dependent variables to be different between groups; see Fig. 1 for the initial unrestricted path model. In a second nested model, we tested whether coefficients for interactions could be set equal for the two groups. In the next step we investigated which path coefficients could be set equal across groups without significantly worsening model fit. Chi square tests were used to compare nested models. In addition, CFI >0.95, TLI >0.95 and RMSEA <0.05 were used as criteria for acceptable model fit [34].

Third, to determine which fine-grained temperament traits were associated with internalizing and externalizing problems, hierarchical multiple regression analyses were performed in SPSS version 19. In both study groups, skewness and kurtosis of internalizing and externalizing problem behavior was between −1 and 1. To minimize the impact of outliers, values were truncated [35] resulting in four outliers in the referred group set to threshold levels based on distributional box plots. The fine-grained temperament traits that correlated significantly (with *p* < .01) with internalizing and externalizing problems across groups, determined by bivariate (Pearson’s) correlations, were used in the linear regression analyses. Linear regression was first conducted in the total clinically referred sample (*n* = 216), to obtain a more robust estimate of these relations in clinically referred children. A second linear regression was done to compare clinically referred and population children. For these analyses, the combined age and gender matched sample (*n* = 230) was used. A dummy for group (0 = general population and 1 = referred) was included in the models together with its interaction with the fine-grained temperament traits in order to test whether associations between temperament traits and problem behavior differed between groups. Using a backward selection procedure, non-significant interaction terms were removed from the model one-by-one until only significant interaction terms remained in the final model. To account for the difference in ethnicity between the groups, a correction for etnicity was used when the regression coefficients changed more than 10% and the significance of the model changed. Lastly, logistic regression analysis was used to determine associations between fine-grained temperament traits and (sub)clinical levels of comorbid internalizing and externalizing problem behavior (with T-scores >61) in the clinically referred children.

## Results

### Temperament Traits in Clinically Referred Children Compared to General Population Children

Regarding the broad temperament dimensions, there were no differences between the clinically referred and the general population children in levels of negative affectivity and surgency. However, the referred children did have significantly lower effortful control than the general population children (Table 2). Regarding the fine-grained temperament traits, within the dimension negative affectivity, differences were found between the groups only in soothability: the referred children had more difficulty to recover from distress than the general population children. Within the dimension surgency, the referred children showed less smiling/laughter than the general population children. Within the temperament dimension effortful control all subscales were significantly different between the groups in the expected direction, with the referred group scoring lower than the population group (Table 2).

**Table 2:** Temperament traits and comparison of clinically referred (N = 115) and general population children (N = 115)

Negative affectivity	3.63 (0.85)	3.45 (0.70)	−1.74	.083
Anger/ frustration	4.07 (1.21)	3.65 (0.99)	−2.92	<.01
Discomfort	3.45 (1.19)	3.56 (1.18)	0.72	.470
Fear	3.24 (1.08)	3.56 (1.22)	2.07	<.05
Sadness	3.53 (1.23)	3.63 (0.87)	0.70	.486
Soothability	4.13 (1.38)	5.13 (1.03)	6.31	<.001*
Surgency	4.45 (0.79)	4.60 (0.59)	1.65	.100
Activity level	4.50 (1.08)	4.15 (0.91)	−2.65	<.01
Approach	4.31 (1.29)	4.14 (0.91)	−1.14	.254
High-intensity pleasure	4.51 (1.38)	4.79 (1.03)	1.73	.085
Impulsivity	3.97 (1.26)	4.21 (0.95)	1.62	.107
Shyness	3.48 (1.57)	3.21 (1.29)	−1.41	.159
Smiling/laughter	4.86 (1.16)	5.51 (0.84)	4.87	<.001*
Effortful control	4.19 (0.71)	5.23 (0.61)	11.81	<.001*
Attentional focusing	3.77 (1.19)	5.21 (0.91)	10.24	<.001*
Inhibitory control	3.47 (1.0)	4.47 (0.90)	7.97	<.001*
Low-intensity pleasure	4.96 (0.96)	5.75 (0.71)	7.13	<.001*
Perceptual sensitivity	5.51 (1.00)	4.58 (1.35)	5.91	<.001*

*Significant at the Bonferroni-corrected significance level of 0.05/18 = 0.0028

### Relations Between Broad Temperament Dimensions and Child Problem Behavior in Clinically Referred Children Compared to General Population Children

There was no curvilinear relation between effortful control and child problem behavior and no evidence for effortful control as a moderator of the relation between negative affectivity and problem behavior, as a nested path model in which coefficients for paths between the three interaction terms and child problem behavior were set to zero did not show a significantly worse fit (*χ* ^*2*^ = 7.66, *df* = 12, *p* = .81).

**Fig. 2:**
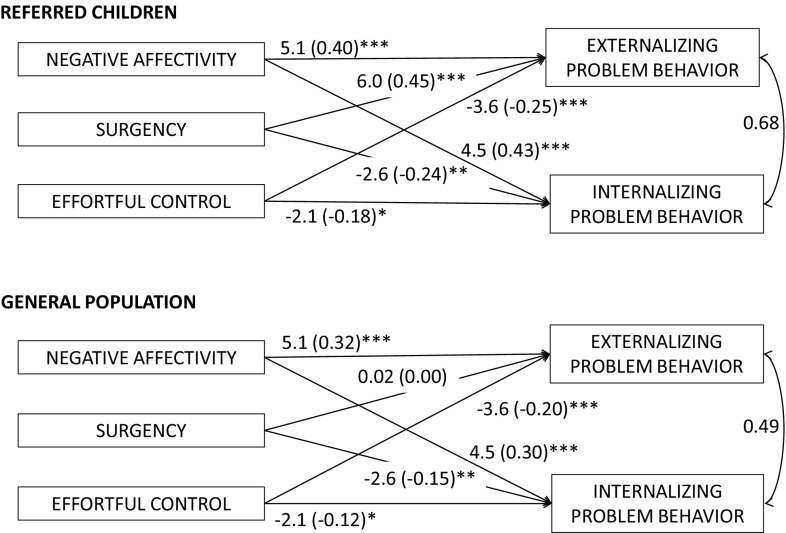
Final models in referred and general population children with unstandardized and standardized (between *brackets*) path coefficients of the relations between temperament dimensions and child problem behavior, *p < .05, **p < .01, ***p < .001

In the final model, only the coefficient for the path between surgency and externalizing problems was allowed to differ between groups (Fig. 2). This finding indicates that the association between surgency and externalizing problem behavior differs between clinically referred and general population children. In the general population group, surgency was not significantly associated with externalizing problems, whereas referred children with higher levels of surgency did show more externalizing problem behavior.

In both groups, higher levels of negative affectivity and lower levels of effortful control were associated with more externalizing problem behavior, with equal magnitude of the associations across the referred and general population group. More negative affectivity, less surgency and less effortful control were associated with more internalizing problem behavior in both groups, with equal magnitude of these associations across the two groups. The final model fitted the data well [χ^2^ = 12.5, *df* = 17, *p* = .77; CFI = 1.00, TLI = 1.03, RMSEA = 0.00 (95% CI 0.00–0.61)].

### Relations Between Fine-Grained Temperament Traits and Child Problem Behavior

#### Externalizing Problem Behavior

The following fine-grained temperament traits were significantly (at *p* < .01) correlated in the expected direction with externalizing problems: frustration, sadness, fear, soothability, high-intensity pleasure, approach, impulsivity, activity level, attention and inhibitory control (Table 3). After correction for internalizing behavior, more frustration, more impulsivity, more activity, less soothability and less inhibitory control were significantly associated with more externalizing problem behavior in the total group of clinically referred children (Table 4). Age and gender did not confound the effects. When comparing referred children and general population children (*n* = 230), there was only a significant difference between the groups in the relation between impulsivity and externalizing problems (regression coefficient of the interaction term *β* = 0.29, *p* < .001). Post hoc analyses in the separate groups showed a positive association for impulsivity in the referred children (*β* = 0.28, *p* = .001), while in the general population children there was no significant association (*β* = −0.02, *p* = .80). Ethnicity did not confound the effects.

**Table 3:** Correlations between fine-grained traits in temperament and child problem behavior in clinically referred children (N = 216) and general population children (N = 115)

Temperament trait	Internalizing problems		Externalizing problems	
	Referred	Population	Referred	Population
Negative affectivity				
Anger/frustration	.45**	.12	.56**	.20*
Discomfort	.10	.09	.00	.18
Fear	.28**	.03	.18**	.08
Sadness	.23*	.04	.22**	.09
Soothability	−.51**	−.35**	−.47**	−.18
Surgency				
Activity level	.01	−.15	.42**	−.02
Approach	.10	.05	.26**	.13
High-intensity pleasure	.06	−.12	.38**	−.01
Impulsivity	−.08	−.22*	.39**	−.12
Shyness	.37**	.34**	.02	.13
Smiling/laughter	−.25**	−.17	−.05	−.12
Effortful control				
Attention focusing	−.06	−.03	−.24**	−.15
Inhibitory control	−.19**	−.05	−.47**	−.12
Low-intensity pleasure	.01	−.08	−.07	−.14
Perceptual sensitivity	.17*	−.04	.05	−.06

*p < .05, **p < .01

**Table 4:** Multiple regression analysis of fine-grained traits predicting externalizing problem behavior, corrected for internalizing problems, in clinically referred children (N = 216)

Fine-grained traits	B	SE (B)	β
Anger/frustration	1.66	.64	.17*
Sadness	−.37	.58	−.04
Fear	−.48	.49	−.05
Soothability	−1.15	.52	−.14*
Activity level	1.65	.67	.17
Approach	.45	.51	.05
High-intensity pleasure	.31	.51	.04
Impulsivity	1.80	.58	.19**
Inhibititory control	−2.66	.66	−.22***
Attention	.13	.53	.01
Internalizing problems	.44	.07	.38***
	Adjusted R² = .60		

*p < .05, **p < .01, ***p < .001

#### Internalizing Problem Behavior

The following fine-grained temperament traits were significantly correlated with internalizing problems: fear, anger/frustration, shyness, sadness, soothability, smiling / laughter, inhibitory control (Table 3). After correction for externalizing behavior, more shyness, less soothability, and less smiling/laughter were significantly associated with more internalizing problem behavior in referred children (Table 5). Age and gender did not confound the effects. When comparing referred children with general population children (*n* = 230), there were no significant differences in regression coefficients between the referred and general population group in the relation between fine-grained temperament traits in relation with internalizing problems. Ethnicity did not confound the effects.

**Table 5:** Multiple regression analysis of fine-grained traits predicting internalizing problem behavior, corrected for externalizing problems, in clinically referred children (N = 216)

Fine-grained traits	B	SE (B)	β
Anger/frustration	1.88	.63	.02
Fear	.46	.47	.06
Soothability	−1.6	.48	−.22***
Shyness	1.86	.38	.28***
Smiling/laughter	−1.3	.52	−.14**
Inhibitory control	.07	.63	.01
Sadness	.37	.53	.04
Externalizing problems	.32	.06	.37***
	Adjusted R² = .48		

*p < .05, **p < .01, ***p < .001

#### Comorbid Internalizing and Externalizing Problem Behavior

Regarding comorbid problems, less soothability (*b* = −0.62, OR 0.54, 95% CI for OR 0.36–0.81, *p* = .003), less inhibitory control (*b* = − 0.72, OR = 0.49, 95% CI for OR 0.28–0.85, *p* = .012) and more frustration (*b* = 0.60, OR 1.82, 95% CI for OR 1.09–3.05, *p* = .023) predicted the presence of (sub)clinical levels of internalizing as well as externalizing problem behavior in referred children. Age, gender and ethnicity did not confound the effects.

## Discussion

This study aimed to examine broad and fine-grained temperament traits and their relation to psychopathology in young clinically referred children, compared to general population children. Regarding levels of temperament traits, results showed that referred children had significantly lower levels of effortful control than general population children, with less capacity to maintain attentional focus and less capacity to plan and suppress inappropriate response reactions. These findings add to earlier research that psychopathology in referred young children may be related to a constitutionally based impaired ability to voluntarily regulate behavior and emotions [29]. Unexpectedly, the referred children did not significantly differ from the general population group in the tendency to react with negative emotions to daily situations, as shown by similar levels of negative affectivity. There was however a difference between the children in levels of soothability (a subscale of negative affectivity). Referred children showed less soothability than general population children. Soothability refers to the rate of recovery after distress, both spontaneously and in response to the soothing techniques parents use. Apparently, the parents of clinically referred children did not notice more intense negative emotions in their children than parents of general population children, but specifically experienced that their children lingered longer in negative emotions, and were more difficult to sooth. In previous population studies, soothability has indeed been found to negatively predict problem behavior in children [24].

Regarding the associations between temperament traits and child problem behavior, we found similarities and differences between referred and general population children. The findings that more negative affectivity and less effortful control each were associated with more internalizing and externalizing problems with associations equal in magnitude for referred and general population children, support the spectrum model [16]. More negative affectivity and less effortful control may well be temperament traits that vary across a continuum and in extreme levels represent psychopathology, as is proposed in other studies [18, 19]. However, we did find a difference between the referred and general population group in the strength of the association between the broad temperament trait surgency/extraversion and externalizing problems. Also, we found a difference between the groups in the relation between the fine-grained temperament trait impulsivity (subscale of surgency) and externalizing problem behavior. The parents of referred children who rated their child as expressing more surgency, and specifically more impulsivity, reported more externalizing problem behavior, whereas this association was not found in the general population group. Possibly, this relation is altered in clinically referred children by a moderating factor such as parenting and/or parent–child interaction [10]. Parents of referred children might react differently than parents of general population children when their children act impulsively, resulting in a stronger association which can add to the risk of developing externalizing problem behavior. Indeed, according to the vulnerability model, specific temperament traits may predispose towards the onset of psychopathology in specific contexts [20]. It is also possible that psychopathology changes the expression of temperament as suggested by the scar model [21] or that parents perceive certain temperament traits of children referred with problem behavior differently than parents of children without problem behavior.

When addressing the relations between fine-grained temperament traits and internalizing and externalizing problem behavior in referred children, several patterns emerged. Within the temperament dimension of surgency/extraversion, there were specific traits (more shyness, less smiling/laughter) related to internalizing problem behavior and other specific traits (more activity and impulsivity) related to externalizing problem behavior. These findings were expected as the traits represent internally focused behavior (i.e., shyness and smiling/laughter) and externally focused behavior (activity and impulsivity). Subscales of negative affectivity and effortful control, namely more anger/frustration, less soothability, and less inhibitory control, were found to be related to clinical levels of comorbid internalizing and externalizing problem behavior. Notably, less soothability was the only trait that was related to more internalizing and externalizing problems when viewed seperately, and also to (sub)clinical levels of comorbid internalizing and externalizing problem behavior. Therefore, soothability might be an important temperament factor in relation to general psychopathology.

Several limitations of this study must be addressed. First, as in most studies investigating young children, only parent reports were used. Although it has been suggested that shared method variance accounts for the association between questionnaire-based assessment of temperament and problem behavior [37], there is also evidence that measurement confound does not account for the association between these constructs as reported by parents at preschool age [8]. Temperament traits have been found to be related to child psychopathology in population studies, even after correction for possible item overlap in questionnaire measures used to assess temperament and child problem behavior [8, 38]. Second, this study used a cross sectional design and therefore no directional or causal conclusions can be drawn. Third, while this study did account for the possible influence of gender, age and ethnicity, we did not investigate other possible relevant factors that might influence the association between temperament and psychopathology. While we did study moderation between temperament traits, previous studies have also shown that temperament traits can function as moderators in the relations between parenting/parental psychopathology and child psychopathology [39]. Strength of this study is that the subscales of broad temperament dimensions were included to find specific relevant fine-grained temperament traits in association with problem behavior, which has often been neglected in previous research [9, 24, 40]. The results of this study show that it is important in research on temperament and psychopathology to focus on fine-grained traits within the broad temperament dimensions. In further research, fine-grained temperament traits in referred children could also be investigated in relation to other specific problem behavior, such as social and attachment problems and outcome of treatment.

Despite the limitations, the main results of this study are in accordance with earlier research and provide implications for early assessment and treatment in young children referred for emotional- and behavioral problems. Self-regulation, including inhibitory control, has already been addressed as an important factor in temperament-based prevention intervention in children [28] and in treatment programs for children with externalizing disorders. The results of this study support this and further suggests that impulsivity is a specifically important temperamental factor in relation to externalizing problems in clinically referred children. When specific temperament traits of children, such as impulsivity or shyness, are recognized by parents and other caregivers, both caregivers and child can learn how to deal with the traits and reduce potential negative consequences. Furthermore, the results of this study suggest that it could also be important to address soothability, the ability to calm down after distress, in diagnostic and treatment interventions of both internalizing and externalizing problems and disorders in referred children. It can be addressed in parent (caregiver)-child treatment, since young children are still dependent on their primary caregivers to help them learn to regulate their emotions and behavior.

### Summary

In the current study, temperament traits and psychopathology were assessed in 216 young clinically referred children. Furthermore, on the same measures, a subset of 115 clinically referred children was compared to 115 age and gender matched children from the general population. Results showed that more negative affectivity and less effortful control may well be temperament traits that vary across a continuum and in extreme levels represent psychopathology in young children. Results also showed that temperamental impulsivity within surgency was more strongly related to externalizing problems in clinically referred children compared to children from the general population, suggesting a vulnerability or a scar effect. Results further suggest that various fine-grained temperament traits are specifically related to internalizing problems and externalizing problems. Meanwhile, soothability was found to be related to both domains of problems and could therefore be a factor related to general psychopathology. We propose that assessment of temperament in clinically referred children may be of help when customizing diagnostic procedures and tailoring treatment interventions in early childhood.
